# Effects of Cold Stimulation on Cardiac-Vagal Activation in Healthy Participants: Randomized Controlled Trial

**DOI:** 10.2196/10257

**Published:** 2018-10-09

**Authors:** Manuela Jungmann, Shervin Vencatachellum, Dimitri Van Ryckeghem, Claus Vögele

**Affiliations:** 1 Institute for Health and Behaviour Faculty of Language and Literature, Humanities, Arts and Education University of Luxembourg Esch-sur-Alzette Luxembourg

**Keywords:** cold stimulation, heart rate reduction, lateral neck region, diving reflex, stress reduction, wearable electronic devices, thermode-based stimulation, acute stress, technology for stress relief, vagus nerve stimulation, psychological stress

## Abstract

**Background:**

The experience of psychological stress has not yet been adequately tackled with digital technology by catering to healthy individuals who wish to reduce their acute stress levels. For the design of digitally mediated solutions, physiological mechanisms need to be investigated that have the potential to induce relaxation with the help of technology. Research has shown that physiological mechanisms embodied in the face and neck regions are effective for diminishing stress-related symptoms. Our study expands on these areas with the design for a wearable in mind. As this study charts new territory in research, it also is a first evaluation of the viability for a wearables concept to reduce stress.

**Objective:**

The objectives of this study were to assess whether (1) heart rate variability would increase and (2) heart rate would decrease during cold stimulation using a thermode device compared with a (nonstimulated) control condition. We expected effects in particular in the neck and cheek regions and less in the forearm area.

**Methods:**

The study was a fully randomized, within-participant design. Volunteer participants were seated in a laboratory chair and tested with cold stimulation on the right side of the body. A thermode was placed on the neck, cheek, and forearm. We recorded and subsequently analyzed participants’ electrocardiogram. The cold stimulation was applied in 16-second intervals over 4 trials per testing location. The control condition proceeded exactly like the cold condition, except we manipulated the temperature variable to remain at the baseline temperature. We measured heart rate as interbeat intervals in milliseconds and analyzed root mean square of successive differences to index heart rate variability. We analyzed data using a repeated-measures ANOVA (analysis of variance) approach with 2 repeated-measures factors: body location (neck, cheek, forearm) and condition (cold, control).

**Results:**

Data analysis of 61 participants (after exclusion of outliers) showed a main effect and an interaction effect for body location and for condition, for both heart rate and heart rate variability. The results demonstrate a pattern of cardiovascular reactivity to cold stimulation, suggesting an increase in cardiac-vagal activation. The effect was significant for cold stimulation in the lateral neck area.

**Conclusions:**

The results confirmed our main hypothesis that cold stimulation at the lateral neck region would result in higher heart rate variability and lower heart rate than in the control condition. This sets the stage for further investigations of stress reduction potential in the neck region by developing a wearable prototype that can be used for cold application. Future studies should include a stress condition, test for a range of temperatures and durations, and collect self-report data on perceived stress levels to advance findings.

## Introduction

### Background

Never before in human history have people been exposed to such fast-paced lifestyles as today, where stress penetrates all areas of everyday life. Stress is a central issue in modern life that affects a broad range of people at various ages and in different professions, beyond levels that are believed to be healthy (eg, [1-3]). Some groups are, however, found to be more vulnerable to stress than others. According to the American Psychological Association [1], groups that are consistently struggling with stress are women and parents, but also younger generations (18-35 years old). In particular, younger people report higher stress levels than any other generation and seem to have difficulties coping with stress [4]. Similarly, a pan-European poll conducted with the general European population showed that 51% of all workers reported that work-related stress was common in their workplace, while 66% attributed stress to hours worked or workload [2]. Additionally, 77% of the European population (36 countries) believed that their job-related stress would increase over the next 5 years [3]. The European Commission has noted the severity of the problem and has made stress and stress management a priority area in Europe’s health strategy for 2014-2020 under the framework of psychosocial risks [5].

This snapshot of psychological stress shows that higher stress levels are quite common in highly industrialized nations. Although the situational context that causes stress for the individual may differ between people, depending on personality characteristics, personal history, etc, physiological responses to perceived stress (eg, elevated heart rate and increased respiration) are consistent indicators across individual stress experiences. Acute stress stimulates the sympathetic nervous system, resulting in the ergotropic activation of the cardiovascular, endocrine, and immune systems [6-8]. Physiological stress responses are highly adaptive in the short term, but with regular elicitation they may become maladaptive and the individual’s activation level turns chronic. Prolonged exposure to stress has been shown to be associated with long-term health consequences that can lead to disease [6-9].

An ever-growing number of individuals are pursuing well-being in their lives by seeking to deploy smart watches and other digital devices in the hope of reaching a better state of health [10]. Wearable devices have been a popular support in social movements that are spearheading physical self-improvement (eg, the quantified self movement [11]). But digitally mediated solutions for stress reduction are rather sparsely forthcoming. Most devices that aim to reduce daily stress experiences track body metrics (eg, heart rate and respiration) and make offline suggestions, such as exercise and meditation, to disengage from stress [12]. As a result, these solutions are limited in their effectiveness because they do not intervene directly at the time of the acute stress experience. They require behavioral changes that, when applied, will lead to stress reduction. Yet behavioral change necessitates a dedicated time investment, which presents a dilemma for individuals who are already struggling with insufficient time in their day [13,14]. Not only a time commitment is needed; some of the suggested solutions also require an undisturbed personal space. This is an additional factor that may make these solutions impractical in everyday settings, where most people are involved with ongoing interactions with other individuals, such as at the office or when taking care of children. Hence, there is a market demand for technologies that provide an approach that not only measures stress but also presents an integral, noninvasive, digitally mediated solution to stress reduction. The solution should provide effective results but also fade into the background of the fast-paced lifestyles that most people experience.

We present an initial study investigating a wearable concept for well-being with the prospect of reducing stress as indexed by increased cardiac-vagal activation. The concept presents an integral solution. It measures an individual’s stress levels using heart rate and computing heart rate variability, and it comprises the strategic placement of a cold stimulus to counteract physiological stress by increasing cardiac-vagal activation.

This study is a first exploration of the effects of cold stimulation in the neck region on heart rate and heart rate variability under resting conditions. Stimulation of the neck region as location on the body and cold temperature as a stimulus have been investigated in previous research on stress reduction, but in separate contexts and on different grounds, for example, vagus nerve stimulation (VNS) and cold water face immersion (CWFI). To the best of our knowledge, cold stimulation in the neck region has not yet been combined into a single study. As this is the first study to investigate the effects of cold stimulation in the neck area on heart rate variability and heart rate, we focused on parasympathetic nervous system activation to clearly assess any potential for heart rate reduction. We accordingly selected a study design that de-emphasized complexity and favored a conservative approach by testing participants under resting conditions.

### The Relationship Between Heart Rate, Heart Rate Variability, and Stress

Heart rate variability is associated with functions of the regulatory and homeostatic autonomic nervous system, which is a key structure in physiological arousal with its sympathetic and parasympathetic nervous system branches. Heart rate and heart rate variability constitute measures of autonomic function, whereas heart rate variability provides more information on the dynamic modulation between the sympathetic and parasympathetic branches of the autonomic nervous system [15].

Research has also suggested that the brain and the heart are connected bidirectionally due to the efferent outflow from the brain affecting the heart and the afferent outflow from the heart affecting the brain [16,17]. The vagus nerve is an integral part of this heart-brain system [18], and heart rate-related measures (eg, heart rate variability) can provide valuable information about the functioning of the heart-brain system. Heart rate variability is the fluctuation of the length of heartbeat intervals [19] and has been suggested to represent the ability of the heart to respond to a variety of physiological and environmental stimuli [20,21].

In recent years, perceived stress levels have been known to be closely associated with measures of cardiac autonomic function, such as heart rate and heart rate variability. For example, Uusitalo et al [22] found that chronic, work-related stress was associated with cardiac autonomic function in hospital nurses at work who were tested over the course of 2 days. Collins et al [23] observed similar findings over a period of 48 hours, studying 30 men who experienced job strain in various job settings. Many other studies (eg, [24-26]) recording short-term or long-term electrocardiograms (ECGs) found comparable results, confirming the association between subjective stress and heart rate variability in larger populations. A recent meta-analysis by Kim et al [27] indicated that heart rate variability can serve as a physiological indicator of stress, which substantiates the findings of an earlier meta-analysis by Thayer et al [28].

Heart rate and heart rate variability are reliable, easily recorded and computed measures. They are assumed to be objective measures of psychological health and stress [27,28] and have often been assessed in cardiovascular and stress research [29-33]. It has also been shown that individuals engaging in biofeedback training are able to control heart rate variability and therefore influence their perception of stress and anxiety [34-36].

In the research reported here, low physiological arousal, or relaxation, was associated with low heart rate and high heart rate variability, indicated by predominately parasympathetic nervous system activation. High physiological arousal, such as that caused by the experience of stressors, indicates high heart rate and low heart rate variability with prevailing sympathetic nervous system activation [37-39]. From the perspective of a wearable concept for stress reduction, heart rate measurements and heart rate variability calculations are already implemented in commercial wearable devices such as chest-worn ambulatory heart rate monitors (eg, heart rate chest belts by Polar [40]). The technology of these heart rate monitors is mature; it has been tested for accuracy and robustness (eg, [41-47]) and can be engineered as part of the design of a stress reduction device.

### Vagus Nerve Stimulation and the Diving Reflex

Cardiovascular research has identified physiological mechanisms that promote relaxation in patients with different physiological and psychological disorders. Methods for heart rate reduction are equally of interest to athletes, who need to speed up their return to homeostasis after physical exertion (eg, [48,49]). Various scientific disciplines, such as sports science and the neurosciences, have studied these physiological mechanisms that initiate relaxation, but their relationship and functional purpose in the body have yet to be fully explicated (eg, [50,51]).

The physiological mechanisms involved are predicated on cranial nerves that are responsible for cardiac-vagal activation and are typically located in the facial area and the head and neck regions. The ascending pathways of the cranial nerves, for example the vagus nerve, transmit various interoceptive signals to the brain (eg, temperature, pain, and pressure), and the descending pathways regulate the functioning of inner organs.

VNS and the diving reflex are the most relevant and accessible physiological mechanisms in relation to our wearable concept. VNS has been used for decades as a therapeutic option in refractory epilepsy. As a method, it has been continuously refined so that applications have broadened to wider patient populations addressing, for example, migraine headaches, Alzheimer disease, depression, and treatment-resistant anxiety disorder [51,52]. VNS is applied to counteract sympathetic nervous system activation by increasing activation of the parasympathetic nervous system, which then induces relaxation in the patient. Direct VNS necessitates the surgical implantation of electrodes in the patient’s chest region with extensions threaded under the skin that attach to the cervical vagus nerve in the neck region. Through the implant, electrical impulses are delivered to the vagus nerve that lead to activation. Newer medical VNS applications cause fewer side effects than their invasive VNS counterpart because they deliver electrical impulses through the skin. There are two approaches, termed transcutaneous VNS and noninvasive VNS.

These newer applications either stimulate the auricular branch of the vagus nerve on the external ear through an intra-auricular electrode, or they use metal disks to make contact with the skin on the neck for stimulation of the cervical vagus (commercial examples are Nemos [53] and GammaCore [54]). Transcutaneous VNS and noninvasive VNS are officially classified as noninvasive applications; however, all types of VNS devices use low-voltage electrical signals that aim to produce neurological stimulation.

The efficacy of transcutaneous VNS and noninvasive VNS has been analyzed in studies with patients and healthy individuals to better understand the mechanisms involved [55-58]. Still, there are differences between the cervical vagus and its auricular branch, which need to be taken into account when assessing the vagus nerve’s overall potential for stress reduction. Nonis et al [59] conducted an experiment with 12 healthy volunteers, comparing noninvasive VNS with transcutaneous VNS applications. In the control condition for each approach, the stimulation was applied to the long muscle of the neck (sternocleidomastoid muscle). Measuring the electrical activity of the brain (somatosensory evoked potential) during stimulations, the researchers found that cervical noninvasive VNS elicited a reproducible response of vagal afferent activation in 11 of the 12 healthy volunteers, whereas stimulation of the auricular branch evoked a comparable response in only 9 of the 12 healthy volunteers. Morphological studies may explain the predominant response of the cervical vagus nerve. Verlinden et al [60] analyzed 11 pairs of cervical vagus nerves and 4 pairs of intracranial vagus nerves with computer software. They found that the right cervical vagus nerve on average had a 1.5 times larger effective surface area than the left vagus nerve. They also showed that the right cervical vagus nerve contained on average 2 times more tyrosine hydroxylase-positive nerve fibers than the left nerve, which can positively influence stimulation.

These results informed our study design. We justified the placement of the cold stimulus during experimentation on the cervical vagus nerve, choosing the right lateral neck area to maximize the potential for a parasympathetic nervous system response.

The diving reflex in humans has been characterized by a pattern of respiratory, cardiac, and vascular responses. Some researchers believe that the diving reflex’s primary role is to ensure survival when diving into water by conserving oxygen, although there is no consensus on this explanation. A potent stimulus to induce the diving reflex is water contact on the forehead, cheek, eyes, and nose. Trigeminal-brainstem-vagal pathways supply these areas, and stimulation inhibits respiration and provokes vasomotor centers and cardiac-vagal motoneurons [61-65].

In particular, the (cold) temperature aspect of water causes superficial cold receptors to be innervated by the ophthalmic branch of the trigeminal nerve, which promotes the cardiac-vagal activity of the diving reflex [66,67]. De Oliveira Ottone et al [48] studied 8 active men who exercised at submaximal levels and subsequently underwent a 15-minute recovery period in a water tank with cold water at 15°C, and hot water at 28°C and 38°C. The results indicated that CWFI accelerates while hot water immersion blunts postexercise parasympathetic activation.

Cold water immersion may also be effective at short time intervals. Buchheit et al [68] reported that cold water immersion for 5 minutes at 14°C after 10 male cyclists performed submaximal aerobic fitness exercise produced faster parasympathetic reactivation than the control condition. Heindl et al [69] studied the effects of ice cubes on heart rate and blood pressure in 9 healthy volunteers who self-administered the stimulus on forehead, hand, and nasal cavities at 2.5-minute intervals alternated with a 10-minute rest. The bronchial system was also cooled down via cold air at –25°C. Heart rate was significantly reduced only during cooling of the nasal cavities and forehead. In sum, cold stimulation applied in short intervals is a promising candidate for parasympathetic activation, and we incorporated it into our study design.

The diving reflex was previously assumed to be strongly linked to breath holding, but recent research suggested otherwise. Kinoshita et al [70] conducted an experiment with 8 healthy volunteers to observe the effects of CWFI and warm water face immersion with and without breathing on heart rate and heart rate variability. The results showed that CWFI diminished cardiac output and increased vagal activity independent of change in body position caused by bending over a basin and unrelated to breath holding. The findings on breath holding confirmed earlier research by Hayashi et al [71], who tested 15 healthy volunteers in 12 trials over 2 days on CWFI with and without breathing. The researchers found that the diving reflex without breath holding increased heart rate variability significantly, indicating that CWFI alone increases vagal activity.

Based on the reviewed research outcomes, we investigated the effects of cold stimulation of the right lateral neck region using a thermode instrument. Control body locations selected for the study were the cheek and forearm. We chose the cheek region because it is central to the diving reflex, and we expected our results to reflect activation of the diving reflex. As the forearm has no known sympathetic or parasympathetic innervation, we did not expect any changes to heart rate or heart rate variability from baseline.

Potential mechanisms underlying any effects of cold stimulation in the neck region are difficult to predict because, in both cranial nerves (vagus and trigeminal nerves), signal relay involves the brainstem regions that indirectly or directly influence the neurochemistry of large areas in the central nervous system [72].

### Objective

Noninvasive VNS is a medical procedure that reduces stress symptoms by lowering heart rate and increasing heart rate variability in patients with serious illnesses. This is achieved through low-voltage electrical impulses sent to the cervical vagus nerve. However, when addressing the general public, the use of electrical impulses as a preventive measure for acute stress is not a viable option without medical supervision.

A safer alternative is cold stimulation, which has been used elsewhere to trigger relaxation (eg, [48,49,67,68,70,71]). The overall aim of this project was to design a technique that is effective as a relief strategy for acute stress and can be deployed by otherwise healthy individuals. The technique combines the placement of the stimulus along the cervical vagus (informed by noninvasive VNS) with cold stimulation (referenced from research on the diving reflex).

The objective of this study was to address the following hypotheses: (1) that cold stimulation of the neck and cheek (but not the forearm) should result in higher heart rate variability than in the (nonstimulation) control condition, and (2) that cold stimulation of the neck and cheek (but not the forearm) should result in decreased heart rate compared with the (nonstimulation) control condition.

## Methods

### Recruitment

Participants were 71 healthy volunteers (43 female), with a mean age of 26.8 years (SD 8.57). Volunteers were university students and staff from Luxembourg University. Exclusion criteria were self-reported chronic physical and mental health conditions (eg, bronchial asthma, cardiovascular disorders, depression) or current acute illnesses (eg, flu) and medication intake with known effects on autonomic nervous system function. We further excluded participants with addictions (eg, alcohol, nicotine), as well as pregnant women. We assessed participants’ health status via a telephone interview prior to the study. All participants gave informed consent at the start of the study, which was approved by the university’s Ethics Review Panel. On completion of the study, participants were compensated for their time and effort in the form of gift vouchers (€10).

### Study Design

**Figure 1:**
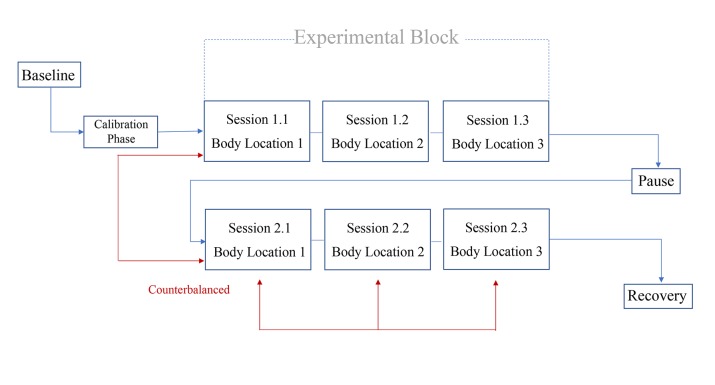
Schematic breakdown of the experiment: each session consisted of four 16-second trials at one body location and 16 seconds at baseline temperature after each trial. The duration of baseline and recovery periods was 3 minutes, and the rest period (pause) between experimental blocks was 5 minutes.

The design was a fully randomized, within-participant study. Participants were presented consecutively with cold and neutral stimuli through a thermode device (PATHWAY Model ATS Pathway System, Medoc Ltd, Ramat Yishai, Israel) on 3 body locations (neck, cheek, and forearm). The order of presentation was counterbalanced over participants (see Figure 1), resulting in 6 configurations (see Table 1). Each participant followed the same configuration order throughout the experimental blocks. An experimental block is defined as 3 sessions, 1 for each body location (neck, cheek, and forearm). Each participant engaged in 2 experimental blocks (cold stimulus and control condition), whereby the order of the blocks was counterbalanced from one participant to the next. A total of 35 participants received the cold stimulus condition first followed by the control condition, and 36 participants completed the study in inverse order. Participants individually attended a single experimental session at the university’s psychophysiology laboratory. Prior to the main study, we conducted a pilot study with 18 participants.

#### Calibration Phase

**Figure 2:**
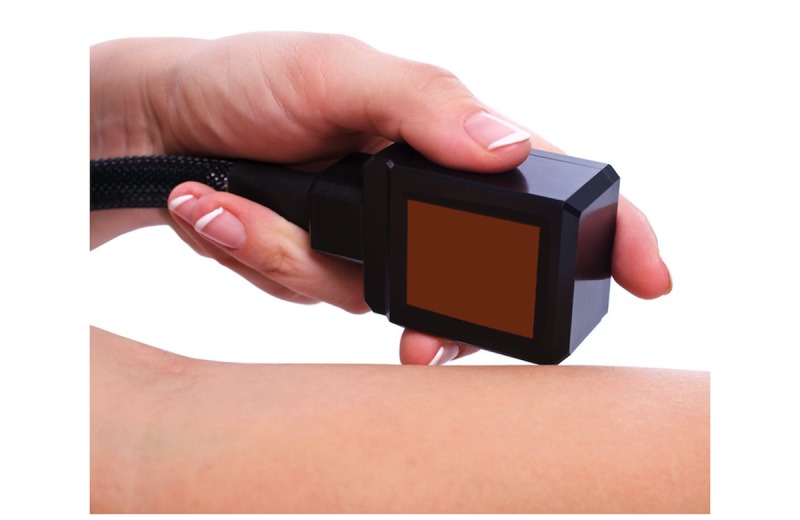
Thermode head used for stimulus application during the sessions. Cold is transmitted only via the 3×3-cm surface (dark red) of the head.

During the calibration phase, we assessed cold sensitivity by determining whether the preselected temperatures were appropriate for the individual participants (see Figure 1 and Figure 2). Final temperatures were scaled by age and gender and adjusted after a pilot study with 18 participants. During the pilot sessions, we used the temperatures indicated in Table 2. Cold temperatures were determined by corroborating indexes from pain studies (eg, [73]) and studies on postexercise recovery experiments using CWFI (eg, [49,68]). After collecting all the data from the pilot sample, we adapted the temperatures on the basis of participants’ input (see Table 3). Among the participants in the pilot sample, 7 considered the temperatures too cold. We then made adjustments to accommodate a larger number of participants and to ensure that temperatures were perceived as not unpleasant. During the calibration period, we marked thermode positions on the participant’s skin to ensure that the placements remained identical when repeating trials.

**Table 1:** Order configurations for cold stimulus and the control condition on neck, cheek, and forearm. Assignment of orders was randomized.

Applied configurations	Sequence of stimulus on body locations
1	Cheek, neck, forearm
2	Cheek, forearm, neck
3	Neck, forearm, cheek
4	Neck, cheek, forearm
5	Forearm, neck, cheek
6	Forearm, cheek, neck

**Table 2:** Initial temperature ranges used for participants (N=18) in the pilot sessions.

Group by age and body location		Temperature by gender (°C)	
		Female (n=14)	Male (n=4)
**Adults <40 years old**			
	Cheek	16	13
	Neck	16	13
	Forearm	14	10
**Adults >40 years old**			
	Cheek	10	9
	Neck	10	9
	Forearm	8	5

**Table 3:** Adjusted temperature ranges used for participants (N=71) of the main sample during the experimental sessions.

Group by age and body location		Temperature by gender (°C)	
		Female (n=43)	Male (n=28)
**Adults <40 years old**			
	Cheek	18	17
	Neck	19	18
	Forearm	18	17
**Adults >40 years old**			
	Cheek	18	16
	Neck	19	17
	Forearm	18	16

#### Stimulus Application

The experimental block consisted of 3 sessions with 4 trials at 1 body location, adding up to 24 trials for the entire course of the study. Each set of 12 trials comprised either the cold stimulus or control conditions for the neck, cheek, and forearm. Trials carried out in the control condition used the baseline temperature of 31°C during the thermode application. The control condition proceeded in identical manner to the cold stimulation. Any differences in responses were therefore due to temperature stimulation and not, for example, tactile stimulation due to the thermode being placed onto the skin.

**Figure 3:**
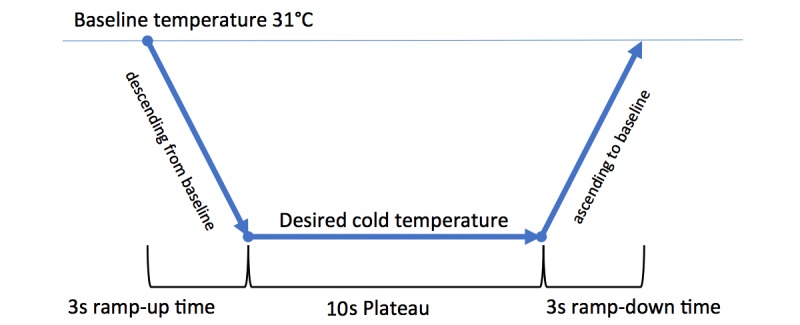
Schematic depiction of the thermode’s temperature change during stimulus application.

Stimulus duration was 16 seconds in total and consisted of a 3-second ramp-up period from baseline (31°C), a 10-second leveling-off period, and a 3-second ramp-down period back to baseline (Figure 3). The 16-second stimulus was succeeded by 16 seconds at the baseline temperature of 31°C. Participants were instructed to focus their gaze on a cross displayed on a computer screen that changed from white to green when the stimulus was active. The sequence of body locations was communicated to the participant via the monitor screen.

### Procedure

On arrival, participants gave informed consent and were generally informed about the upcoming procedure but were not made aware of the research hypothesis. Participants were then led into a separate chamber inside the laboratory room, where they were seated in an armchair, facing a computer monitor with a computer mouse within their reach. Transducers were attached in an Einthoven lead-II position for ECG monitoring. A baseline of a 3-minute continuous ECG was recorded under resting conditions. During this time, participants were unattended and instructed to fixate their eyes on a white cross displayed on a computer monitor located in front of them.

After the baseline was recorded, participants started with the first experimental block. To induce cold stimuli, we used a thermode with a 3×3-cm head. All stimulus and control applications were placed only on the right side of the body. Throughout the experimental blocks, 2 research assistants trained in the experimental procedure handled and held the thermode stationary onto the designated places of the participant’s body. The research assistants were oblivious to the research hypotheses and to any temperature ranges or shifts in the thermode while holding the device (see Figure 2).

The cold stimulus for the cheek was positioned in the middle of the participant’s right cheek. For the cold stimulus on the right lateral neck, we selected a place inside the posterior triangle near the clavicle head. The cold stimulus applied to the forearm was placed on the right outer forearm, halfway between the right hand and elbow.

Between experimental blocks, the participant was moved from the chamber to the main laboratory room where he or she sat down unattended for 5 minutes. Heart rate recording was paused during this time, but electrodes remained attached to the chest region. After the pause, the participant returned to be seated in the chamber, and the study advanced with the second experimental block.

**Figure 4:**
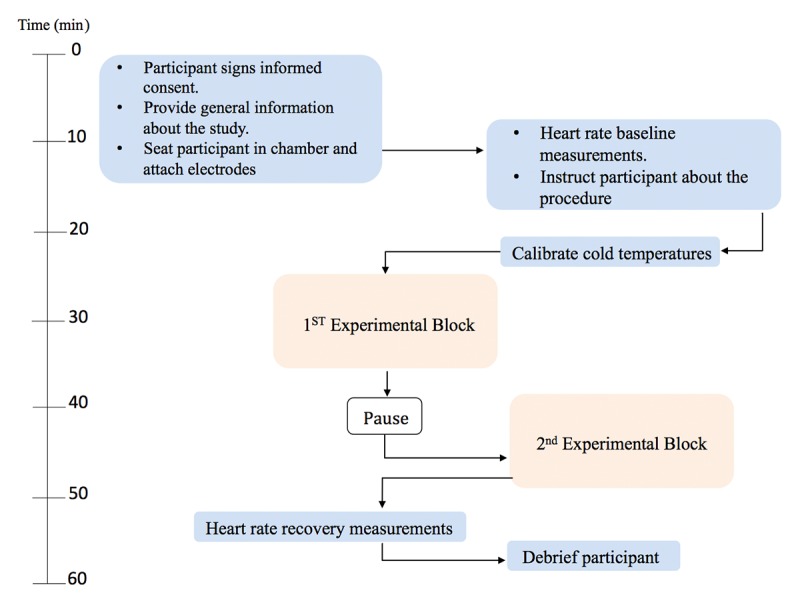
Timeline for the randomized controlled trial. The duration of the experimental blocks and the pause was calculated at 20-25 minutes to compensate for the time of thermode placement on the different body areas and for restabilization of the electrocardiographic signal.

Once the trials of both experimental blocks were completed, a 3-minute recovery period was recorded. As with the recording of the baseline at the beginning of the study, during the recovery recording, the participant was also left unattended. When the recovery period was completed, the research assistants removed the electrodes and the markings from the participant’s body. After being compensated, the participant left the laboratory. Figure 4 depicts the overall timeline of the study procedure.

### Data Collection

#### Equipment and Materials

We recorded the participants’ ECG at a sampling rate of 256 Hz using a Biopac MP150 amplifier and data acquisition system with AcqKnowledge software version 5.0 (Biopac Systems, Inc, Goleta, CA, USA). ECG was continuously recorded throughout the experimental blocks, as well as at baseline and during recovery periods. We programmed the experiment in E-prime (Psychology Software Tools, Inc, Sharpsburg, PA, USA), which triggered the Medoc thermode (Figure 2) and presented instructions on a computer screen. The ECG signal was stored on a hard disk. Beat detection and artifact control was performed offline with WinCPRS software (Absolute Aliens Oy, Turku, Finland).

The R-wave peak was detected automatically and was followed by a manual correction step where QRS complexes in some cases were not detected by the automatic algorithm and we had to set it manually. We based the automatic R-wave peak detection on application of a distribution-related threshold criterion that we adjusted individually for each participant. As the data showed no ectopic beats or arrhythmia, no other artifact control was necessary. Time domain measures were directly calculated from R-R interval series.

#### Data Reduction and Statistical Analysis

We expressed heart rate as interbeat intervals (IBIs) measured in milliseconds. We separately averaged IBIs from baseline, cold stimulus, and control condition trials, as well as recovery values. We analyzed heart rate variability from the IBI in ultrashort-term heart rate variability analysis. The time intervals had a duration of 64 seconds, each comprising 4×16 seconds per body region (neck, cheek, and forearm). We analyzed heart rate variability using WinCPRS.

As the heart rate variability index, we used the root mean square of successive differences (rMSSD), which is the standard time domain measure for detecting autonomic nervous system activation, in particular parasympathetic activity in short-term measurements.

rMSSD is correlated with the vagus-mediated components of heart rate variability [74] and has better statistical properties than other metrics, such as the proportion of the number of successive N-N intervals that differ by more than 50 milliseconds divided by the total number of N-N intervals [75]. rMSSD has been found to be the most reliable metric for ultrashort-term heart rate variability analysis, especially for 10-second intervals under resting conditions [76-80]. We excluded as outliers rMSSD and IBI data with more than 3 SD above the mean for each possible combination of location and condition, reducing the sample included in the analysis to n=61.

We analyzed data using a repeated-measures ANOVA (analysis of variance) approach with 2 within-participant factors: body location (neck, cheek, forearm) and condition (cold, control). We conducted follow-up pairwise comparisons using paired *t* tests, with the Sidak method used to correct for multiple comparisons. All statistical analyses were carried out with IBM SPSS 24 Statistics (IBM Corporation).

## Results

### Sample

The participants’ ages ranged from 19 to 51 (mean 26.8, SD 8.57) years. Age was nonnormally distributed with a skewness of 1.16 (SE 0.28) and kurtosis of 0.40 (SE 0.56). Women accounted for 60% of the sample.

### Heart Rate Variability

A first exploration of the rMSSD data showed skewness and kurtosis in the ranges of 1.71 (SE 0.28) to 2.63 (SE 0.28) and 2.01 (SE 0.56) to 8.26 (SE 0.56), respectively, indicating deviations from the normal distribution. To normalize data, we transformed the data using log transformation (log base 10).

A 2×3 ANOVA on rMSSD with condition-by-body-location trials (cold and control condition at neck, cheek, and forearm) as within-participant factors revealed a main effect for condition (*F*_1,60_=14.68, *P*<.001, η_p_^2^=.20), demonstrating that heart rate variability across body locations differed significantly between conditions. Throughout the stimulation sites, heart rate variability was significantly higher during cold stimulation (mean 1.04, SD 0.25 ms) than in the control stimulation (mean 1.01, SD 0.24 ms). Furthermore, the main effect for body location was marginally significant (*F*_2,120_=2.52, *P*=.08, η_p_^2^=.04). Pairwise comparisons indicated that heart rate variability at the neck (mean 1.03, SD 0.25 ms) was marginally higher than at the forearm (mean 1.01, SD 0.25 ms) stimulation site (*P*=.07). No other comparison was significant.

**Figure 5:**
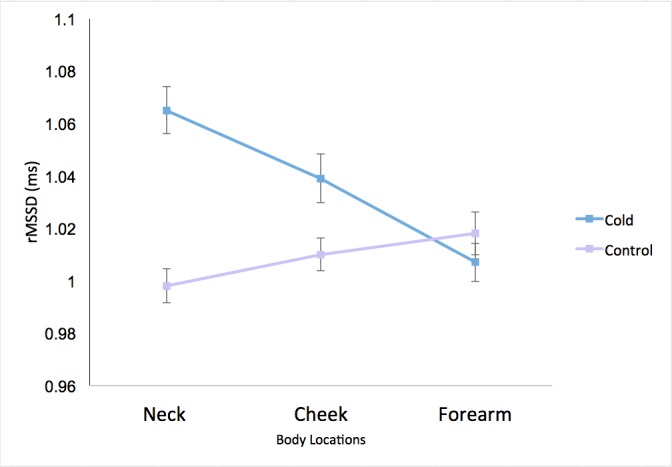
Normalized root mean square of successive differences (rMSSD) of all body locations for cold stimulus and control conditions (n=61). The error bars depict within-participant standard error following Cousineau-Morey corrections [81].

The interaction effect for condition and body location was also significant (*F*_2,120_=21.37, *P*<.001, η_p_^2^=.26). Follow-up *t* tests showed that the experimental and control conditions differed only for neck and cheek, but not for forearm. Cold stimulation on the neck led to higher heart rate variability (mean 1.07, SD 0.26 ms) than in the control condition (mean 1.00, SD 0.25 ms; *t*_60_=6.24, *P*<.001). On the cheek, cold stimulation induced higher heart rate variability (mean 1.04, SD 0.25 ms) than in the control condition (mean 1.01, SD 0.24 ms; *t*_60_=2.88, *P*=.006). There was no appreciable difference between cold (mean 1.01, SD 0.26 ms) and control (mean 1.02, SD 0.25 ms) stimulations for the forearm (*t*_60_=–1.15, *P*=.26). Figure 5 shows the interaction effect [81].

### Heart Rate

**Figure 6:**
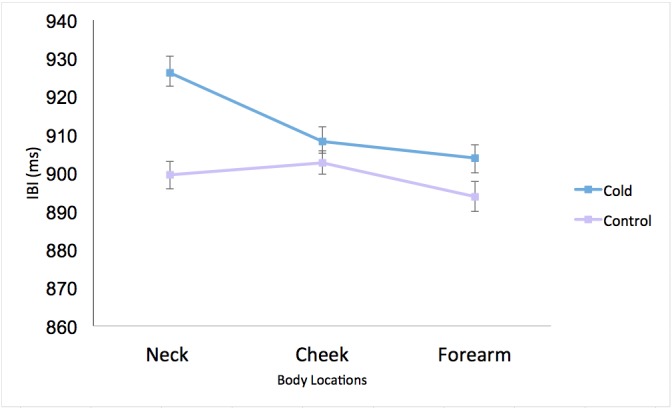
Mean interbeat intervals (IBIs) of all body locations for cold stimulus and control conditions (n=61). The error bars depict within-participant standard error following Cousineau-Morey corrections [81].

We computed a 2×3 repeated measures ANOVA on IBI with condition (cold and control) and body location (cheek, neck, and forearm) as within-participant factors. This analysis revealed significant main effects for condition (*F*_1,60_=9.84, *P*<.01, η_p_^2^=.14) and for body location (*F*_2,120_=7.79, *P*<.001, η_p_^2^=.14). Pairwise comparisons showed significantly longer IBIs in response to stimulation in the neck (mean 912.84, SD 146.69 ms) than in the cheek (mean 905.46, SD 145.06 ms; *P*=.04) region. Similarly, stimulation in the neck area also resulted in longer IBIs than in the forearm (mean 898.87, SD 145.81; *P*=.002). There was no discernable difference in IBIs between cheek and forearm stimulation sites (*P*=.24). The findings, illustrated in Figure 6, show that stimulation in the lateral neck region engendered the lowest heart rate.

There was also a significant condition-by-body-location interaction effect (*F*_2,120_=9.76, *P*<.001, η_p_^2^=.14). As Figure 6 shows, IBIs were longest—that is, heart rate was lowest—during cold stimulation in the neck region (mean 926.25, SD 153.29 ms), as opposed to the control condition (mean 899.43, SD 143.74 ms; *t*_60_=4.42, *P*<.001).

For the forearm stimulation site, IBIs were slightly longer in the cold (mean 903.97, SD 149.14 ms) than in the control condition (mean 893.83, SD 144.82 ms; *t*_60_=2.12, *P*=.04). There were no distinguishable differences in IBIs between the cold (mean 908.23, SD 149.38 ms) and the control (mean 902.7, SD 143.54 ms) conditions for the cheek region (*t*_60_=1.06, *P*=.29).

The overall findings of the study confirmed our main hypothesis that cold stimulation at the lateral neck region would result in higher heart rate variability and lower heart rate than in the control condition.

## Discussion

### Principal Results

This study was motivated by the need for technological support that is effective in reducing acute stress levels in otherwise healthy individuals. With this study, we carried out a first empirical evaluation of the effects of cold stimulation in the lateral neck region on heart rate variability and heart rate. This is an initial step toward assessing the viability of a wearable concept for stress reduction and contributes to the body of basic experimental research conducted toward understanding the effects of temperature stimuli on heart rate and heart rate variability at various body regions. The study design was conservative in that we investigated heart rate reduction and cardiac-vagal activation under resting conditions. To chart this new research territory, which was informed by the combined knowledge from studies in VNS and CWFI, we selected a within-participant approach to collect physiological data in an experimental laboratory setting.

We hypothesized that cold stimulation in the lateral neck and cheek regions would induce higher heart rate variability than in the (nonstimulation) control condition, except for the forearm. Our results confirmed that cold stimulation in the right lateral neck and the cheek areas increases heart rate variability, denoted by higher rMSSD values. These outcomes are in line with previous cold stimulation paradigms, such as CWFI research (eg, [48,49,67,70,71]), which have been shown to modulate parasympathetic activation similar to that observed in the diving reflex. As there have been no previous studies testing for the effects of cold administered to the neck, our findings suggest that the area sensitive to cold potentially extends from the cheek to the neck. Alternatively, cold stimulation in the neck area may have triggered physiological mechanisms known to have an impact in noninvasive VNS. Future studies are needed to investigate the exact physiology underpinning the effects of cold stimulation in the right lateral neck region.

Our hypothesis on heart rate outcomes was only partially supported. Overall, the results showed a pattern of cardiovascular reactivity to cold stimulation as expected; however, we observed this effect only for the neck, but not for the cheek. The heart rate findings regarding the right lateral neck area replicated those observed with heart rate variability. This suggests that cold stimulation in the right lateral neck region has the potential as an effective alternative to electrical impulsing used in VNS interventions.

Unexpectedly, we did not observe any differences in heart rate between the cold and control conditions in the cheek region. Previous research investigating the effects of cold stimulation on heart rate predominately focused on using heart rate variability and the time constant heart rate recovery as postexercise outcome measures (eg, [49,68]). It is consequently difficult to infer from related research the reason behind this contrast in findings.

### Limitations

While the results of our study are promising, there are also limitations. First, the skewed age distribution limits the generalizability of the study’s results. Future studies should strive for an even spread of age when recruiting participants. From the perspective of the wearable, it will be important to confirm the effects of cold stimulation across the whole age range, including the elderly population, because this will determine which market should be targeted for commercialization.

Second, we appropriated a medical device (the thermode) for cold application during the study. It had to be handheld by 2 research assistants at the prespecified location of the participant’s body. Prolonged holding of the thermode may have caused shifts in pressure across body locations and experimental blocks. However, we aimed to minimize any effects by using the counterbalanced study design. Outcomes may have also been influenced by the close physical proximity of the 2 researchers to the participant. However, the distance between research assistants and participants was consistent across experimental blocks.

Third, although a sampling rate of 256 Hz for ECG data collection is a well-established standard in the field (cf [75,82]), future studies may consider using a higher sampling rate to increase the temporal accuracy of heartbeat detection.

### Future Consideration

For this study design, we purposefully omitted a stress condition to perform a first evaluation of the effects of cold stimulation in the lateral neck region on heart rate variability and heart rate. To continue the evaluation, subsequent studies should include a stressor in the study design. A stressor, such as the Trier Social Stress Test, which requires the participant to give a speech in front of a disapproving panel and perform an arithmetic task, would be an effective choice and the next logical step in the evaluation procedure. A follow-up study should also investigate the relationship between heart rate and rMSSD with regard to age and gender by recruiting participants within a wider age bracket.

It would also be advantageous for the continued evaluation to gain insight into the participant’s experience of the cold stimulation. However, we based our choice to exclude self-report ratings in this study on the fact that requesting participants to fill out ratings on the experience of stress repeatedly during the experimental session would have affected our physiological measurement. A follow-up study should collect self-report data on several dimensions, such as perceived stress, relaxation, and pleasantness levels of the cold stimulation placed on the neck.

Since a wearable solution for stress reduction must stand up to the demands of daily living, a field study that assesses the effects of cold stimulation on heart rate variability and heart rate in the neck region in real-life stress situations also suggests itself as a future investigation. A range of cold temperatures and durations should also be included in future research. However, as a thermode would not be suitable for a field study because it is not mobile and cannot be easily fastened to the neck, the development of a basic prototype is required that can be used for cold application under real-life circumstances.

### Conclusion

Our results demonstrated that cold stimulation in the lateral neck region activates the parasympathetic nervous system in ways that resemble significant research findings in VNS and CWFI. For research, it may be of interest to continue this line of investigation, to broaden the scope of VNS and CWFI, which may expand our understanding of the biological mechanisms that underpin parasympathetic activation. For the development of a wearable for stress reduction, this study’s outcomes confirm our hypotheses and substantiate the first step in the evaluation procedure toward the development of a commercial product.

## Abbreviations

ANOVA: analysis of variance
CWFI: cold water face immersion
ECG: electrocardiogram
IBI: interbeat interval
rMSSD: root mean square of successive differences
VNS: vagus nerve stimulation
